# The Valuable Role of Endoscopy in Inflammatory Bowel Disease

**DOI:** 10.1155/2012/467979

**Published:** 2012-02-29

**Authors:** Matthew J. Hamilton

**Affiliations:** Division of Gastroenterology, Brigham and Women's Hospital, Harvard Medical School, Boston, MA 02115, USA

## Abstract

Endoscopy is a valuable clinical tool for the clinician who takes care of patients with inflammatory bowel disease (IBD). The role of endoscopy in the diagnosis, management, and treatment of IBD is discussed in this review. The central role that colonoscopy plays in screening for colon cancer in patients with longstanding IBD is also addressed.

## 1. Introduction

 Endoscopy is an essential clinical tool to assist in the diagnosis and management of inflammatory bowel disease (IBD) [1]. By direct visual inspection of the intestinal mucosa used in conjunction with histology from biopsies, a diagnosis of IBD can be made. Proper use of endoscopy with biopsies also enables evaluation of other disorders that may mimic the gastrointestinal features of IBD. Once the diagnosis of IBD is made, endoscopy is performed to assess the severity and location of inflammation, and to evaluate for other disease possibilities in the midst of a flare. Response to medical treatment or surveillance for postoperative disease recurrence may also be evaluated. Finally, endoscopy is used in colon cancer surveillance in those with longstanding IBD. The role of endoscopy in these areas will be reviewed in this paper. The emerging role of wireless capsule endoscopy and endoscopic ultrasound in IBD will not be addressed.

## 2. Endoscopy in the Diagnosis of IBD

 Because IBD is a chronic lifelong condition that requires careful medical management and followup and can be associated with significant morbidity with hospitalizations and surgeries, establishing that the diagnosis is essential. Once IBD is suspected based on clinical signs and symptoms, laboratory, and/or radiology studies, endoscopy with mucosal biopsies adds considerably to the diagnosis [2, 3]. The index colonoscopy is critical in establishing the disease extent and severity. Special note must be made of the perianal area for tags, fissures, strictures, and fistula tract openings which may suggest Crohn's disease (CD). The rectal mucosa must be carefully inspected and biopsied to evaluate for at least microscopic inflammation as involvement here is always present in ulcerative colitis (UC) [4].

**Figure 1:**
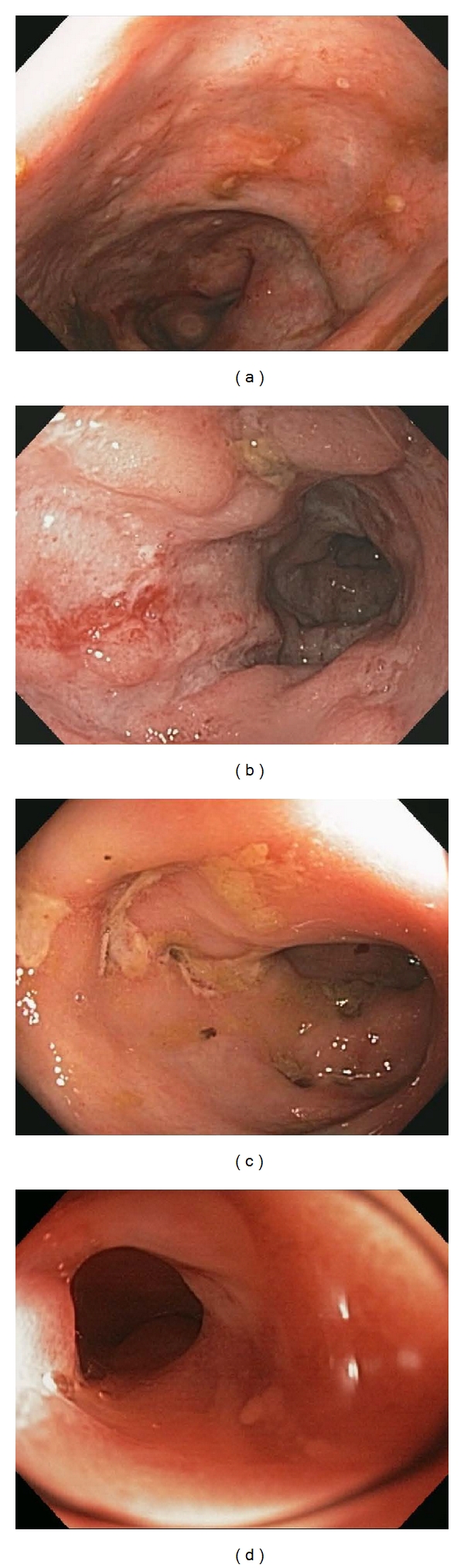
Endoscopic features of Crohn's disease. Several key endoscopic features of Crohn's disease are depicted including apthous ulcers (a), cobblestoning (b), serpiginous ulcers (c), and a stricture in the terminal ileum (d).

**Figure 2:**
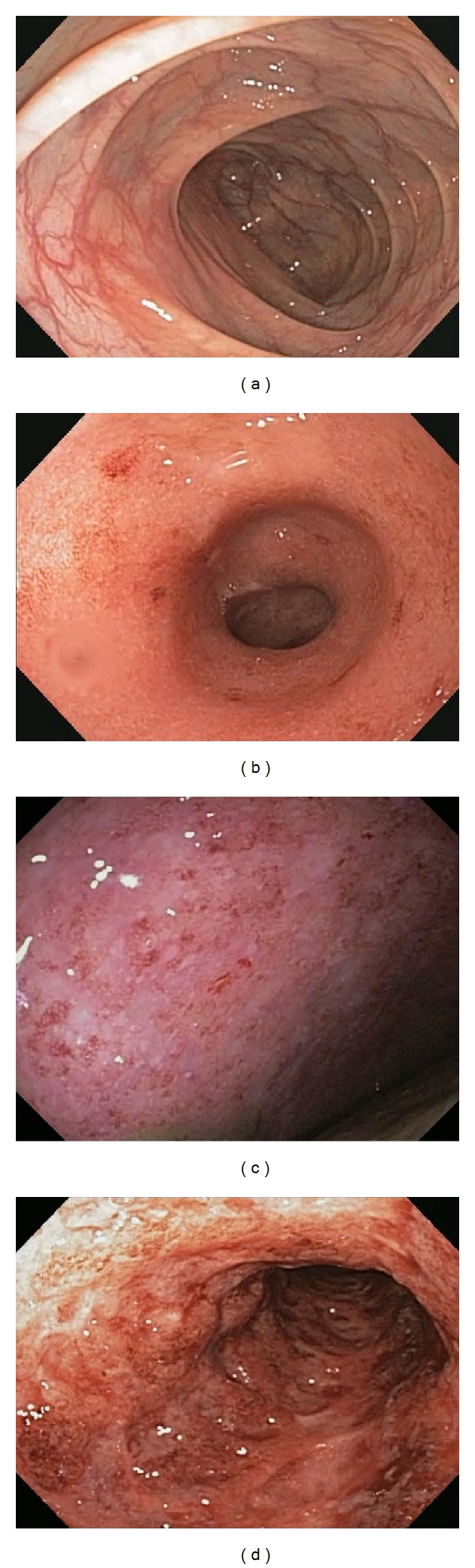
Endoscopic features of ulcerative colitis images of the various degrees of severity of inflammation as assessed by endoscopy are shown compared to a normal colon (a). Mild inflammation is depicted in (b), moderate inflammation in (c), and severe inflammation in (d).

 Close attention to the mucosal features seen throughout the colon can help suggest IBD and even decipher between UC and CD. Sensitive endoscopic features to establish a diagnosis of CD are patchiness of the disease extent, apthous ulcers (Figure 1(a)), and cobblestoning [3] (Figure 1(b)). It is the penetrating nature into the deeper layers of the colon wall that can sometimes give the characteristic cobblestoning appearance. The discrete ulcerations associated with CD are also suggestive of this diagnosis and include linear or serpiginous ulcers (Figure 1(c)). Other features of CD that can be assessed at index colonoscopy include evidence of strictures in the colon, ileocecal valve, or terminal ileum (Figure 1(d)). A colonic stricture in a suspected patient with UC should raise concern for malignancy. Likewise, strictures that are not able to be passed with the endoscope need further evaluation, usually with radiographic imaging. The vascular pattern in CD is also important to note and can represent the patchy nature of CD: normal vasculature might be found next to abnormally inflamed mucosa where the vessels are apparently absent. In UC, it is essential to evaluate and document the extent of inflammation seen which will ultimately help classify the patient as having proctitis (disease limited to the rectum), left-sided disease (disease extends to the splenic flexure), or pancolitis (the entire colon is involved). The severity of inflammation in UC is the other piece of information that is important to note. A mild degree of inflammation as assessed by endoscopy is associated with a loss of the appearance of blood vessels, erythema, and a granular appearance to the mucosa (Figure 2(b)). Moderate disease is associated with mildly erosive disease with increased erythema and edema (Figure 2(c)). Colons with severe disease have ulcers, exudates, and spontaneous bleeding (Figure 2(d)). It may be helpful to use an established grading system in the assessment of the involved mucosa which can then be used to compare future procedures and provide information to the pathologist in a standard manner [5].

**Figure 3:**
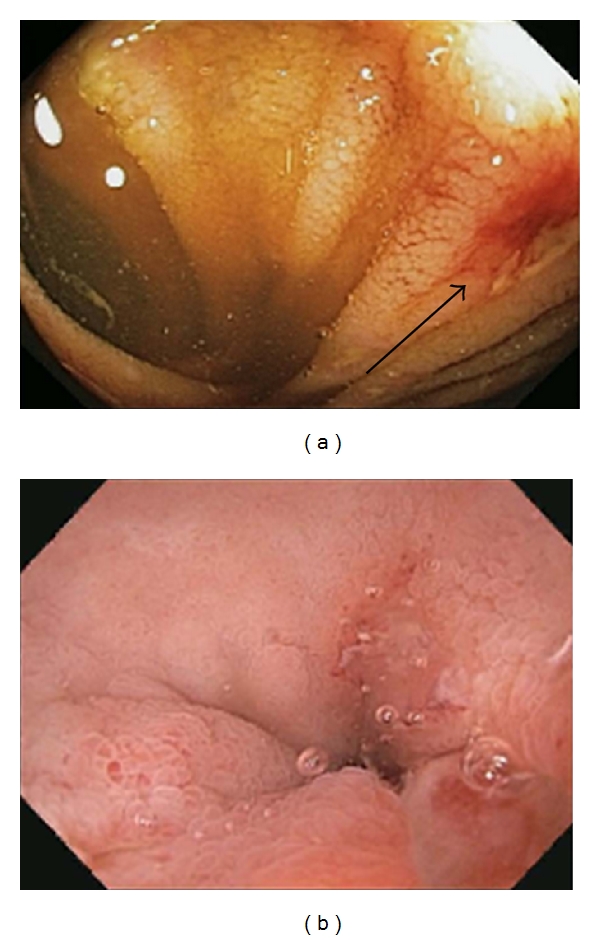
Findings in the terminal ileum. An ulcer (black arrow) is seen 3 cm proximal to the ileocecal valve in a patient with Crohn's disease (a). Erosions in the terminal ileum are shown in a patient who presented with guaiac positive stools and had been taking NSAIDs for back pain (b).

 Ileoscopy is imperative in the initial colonoscopy when evaluating for IBD [6]. Careful inspection of the ileocecal valve for erosions, ulcers, and strictures should be noted prior to intubation of the ileum. In a suspected UC patient without pancolitis, the terminal ileum should be entirely normal. However, if the disease extends through to the cecum, then the appearance of the so called “backwash” ileitis may be noted as erythema and even erosive disease. Ileal involvement in UC was studied in 200 consecutively collected colon resection specimens. In this study, 17% were found to have evidence of ileal involvement [7]. Although the vast majority of these patients had pancolitis (94%), the others did not have cecal involvement. The clinical message from this study is that ileal involvement in a UC patient does not always mean that the diagnosis should be switched to CD. Biopsies of the ileum should always be performed and interpreted using the full clinical context even if the endoscopic appearance is normal [8]. In suspected CD, the terminal ileum should be evaluated for erythema, erosions, ulcers (Figure 3(a)), and strictures. It is important to keep in mind and to note whether or not a patient has been taking NSAID medications when the terminal ileum findings are ultimately documented and interpreted. Prior usage of NSAIDs can lead to identical endoscopic findings as those seen in CD (Figure 3(b)).

 There are several other points to consider regarding the index colonoscopy. One must always assess the relative safety of a full colonoscopy and ileoscopy in those that have moderate-to-severe inflammation, significant pain prior to the procedure, or other medical relative contraindications to the procedure. In these instances a more limited flexible sigmoidoscopy may still allow for some mucosal visualization and allow for biopsies in suspected UC and colonic CD. Radiologic studies may be helpful in right-sided colonic CD or ileal disease. Once a patient has been started on treatment and the severity of disease is presumed to be less and the patient is without pain, then a full colonoscopy with ileoscopy can be performed safely. It is important to keep in mind, however, that medical treatment can potentially change the natural endoscopic appearance of the patient's colon [9]. Topical therapy in the form of suppositories or enemas can yield relative sparing of the rectum and/or left colon, respectively. Systemic medications have also been known to cause a “patchy” distribution of endoscopic inflammation that may be misinterpreted as a classic feature for CD. Biopsies of involved and uninvolved areas of colon and performing the colonoscopy prior to the institution of medical therapy will help avoid these potential pitfalls in diagnosis. Disease that is *de novo* also might not possess the classic endoscopic features of IBD. Newly diagnosed UC may be patchy for instance and have relative or absolute rectal sparing [10]. Similarly, severe UC may possess many of the endoscopic features we associate with CD such as deep, fissuring ulcers, patchy distribution, and ileal involvement [11]. Lastly, UC patients even in the absence of pancolitis may have involvement of the periappendiceal area—the so-called cecal patch [12]. Although this has been found to have no clinical or prognostic significance in these patients, it should be noted and not confused with a diagnosis of CD.

 There are several other conditions that may mimic the clinical signs and symptoms of IBD and a colonoscopy should always be considered for the diagnosis [13]. The endoscopic features of these entities alone may overlap and be nonspecific, and so biopsies and the clinical scenario are necessary to factor in. Infectious colitis is the most common entity that can present like IBD and cause features such as abdominal pain, bloody diarrhea, and terminal ileal disease. In one older prospective study of patients presenting with acute hemorrhagic colitis-type symptoms, infectious colitis was found to be the cause in 38% of the cases [14]. It is important to obtain stool cultures and *c. difficile* studies prior to, or at, the time of colonoscopy to aid in the diagnosis of infectious colitis. Tuberculosis is a consideration when a narrowed, nodular ileum and/or characteristic destruction of the ileocecal valve [15] is encountered, and the appropriate studies should be obtained if tuberculosis is suspected. In infectious colitis, biopsies will not show the histologic features of chronicity such as architectural distortion, basal plasmacytosis, Paneth cell hyperplasia, or pyloric gland metaplasia [16]. Ischemic colitis may have a patchy distribution with varying endoscopic involvement but usually is localized to characteristic locations at the splenic flexure or right colon and is found in an at-risk patient. Biopsies in involved and uninvolved areas will confirm this diagnosis. Similarly, the colitis associated with diverticulosis will be patchy and confined to an area populated by diverticula. The presence of diverticula should be relayed to the pathologist to help with their interpretation. Radiation colitis can be seen in an at-risk patient, and certain medications especially NSAIDs can yield any of the characteristic lesions seen on endoscopy in IBD [17]. The use of these potentially offending medications should always be noted. Finally, microscopic colitis despite its name may be associated with mild endoscopic changes such as erythema and edema, and its diagnosis should be interpreted carefully in conjunction with the biopsy results and clinical presentation.

 There may be a role for upper endoscopy in the patient initially suspected of having IBD, particularly in the young patient with upper intestinal symptoms. Up to 13% of patients with CD may have involvement of the gastrointestinal tract proximal to the ligament of Treitz [18], with the esophagus, stomach, and duodenum all having been reported. Endoscopic features may include erythema and granularity, nodules, and bleeding. Histology may show characteristic findings of chronic inflammation such as architectural distortion combined with a chronic inflammatory infiltrate and granulomas [19]. Upper endoscopy with biopsy might also be useful to rule out other causes of symptoms such as celiac disease, peptic ulcer disease, and eosinophilic diseases. Interestingly, the duodenum of patients with UC has also been shown to be involved endoscopically and does not necessarily support a diagnosis of CD [19, 20].

 Additional small bowel evaluations if indicated can include capsule and double-balloon enteroscopy. These modalities will not be discussed here and were recently reviewed elsewhere [21].

## 3. Endoscopy in the Management of Established IBD

 Once a diagnosis of IBD has been established, endoscopy is an important tool to evaluate response to therapy, postoperative recurrence, new worrisome symptoms, or contributing factors in a flare of the disease. Increasing evidence suggests that mucosal healing is the optimal end point for medical treatment both in UC and CD [22]. In UC, mucosal healing leads to clinical remission and potentially lessens the colon cancer risk overtime [23]. In CD, a prospective study showed that mucosal healing results in clinical remission, and reduced surgeries and hospitalizations at one year [24]. Endoscopy when combined with biopsies will make this assessment, particularly when there can be a poor correlation between the physician's perception of how well a patient is doing and the actual degree of endoscopic inflammation [25]. Colonoscopy is indicated in IBD when there are refractory or breakthrough symptoms to the established medical treatment. In the patient with persistent pain and loose stools for instance, endoscopy can determine whether or not the symptoms are due to active inflammation. For the same reason, it is important to perform a colonoscopy when a new medical treatment is being considered. If there are any new symptoms or signs such as worsening iron deficiency, new bleeding weight loss, or fevers, endoscopy should also be contemplated. If surgery is planned for either UC or CD, a presurgery colonoscopy and ileoscopy is helpful for the surgeon to help decide on the appropriate type and extent of surgery.

 Colonoscopy and ileoscopy is playing an increasing role in the postoperative management of CD, particularly in those who have undergone ileocecal resection. The endoscopic appearance 6–12 months after the surgery [26] can help determine the risk of relapse of disease and decide who should be on aggressive postoperative treatment [27]. Patients who have no disease or only a few scattered apthous ulcers in the neoterminal ileum may likely be watched safely, whereas patients with more severe endoscopic disease should likely start medical therapy.

 Endoscopy is also essential in the IBD patient who has undergone ileal pouch anal anastomosis (IPAA). UC patients are at risk for pouchitis that can lead to abdominal pain, loose stools, and rectal bleeding, and the characteristic features can be noted on endoscopy (usually performed with an upper endoscope). The pouch can appear erythematous, ulcerated, exudative, and with varying degrees of bleeding. Along with the direct endoscopic visualization, biopsies can aid in making this diagnosis [28]. Careful inspection of the afferent limb may reveal a small degree of inflammation at the distal most aspect suggestive of backwash and should otherwise be normal, and this should be confirmed with biopsies. The differential diagnosis of pouchitis includes CD, and this can be seen endoscopically with the characteristic CD lesions in the pouch and involving the afferent limb (not just the distal most aspect). Cuffitis in those with a small amount of retained rectal tissue is important to diagnose with endoscopy and biopsy. As in other IBD patients, one can evaluate for infections including *c. difficile*. Lastly, irritable pouch syndrome is a diagnosis that can be made in the symptomatic patient whose pouch looks endoscopically otherwise normal [29].

 Endoscopy is an important clinical tool in the IBD patient during a flare of disease. As mentioned, endoscopy will be able to determine fairly quickly if the new symptoms are secondary to active inflammation. Endoscopy and biopsy will also help to differentiate a true flare of IBD versus a complicating infection such as *c. difficile* or CMV. It is important to note that the appearance of *c. difficile* in the IBD patient may lack pseudomembranes and be difficult to distinguish from IBD alone without the aid of stool toxin studies [30]. The key diagnostic finding in CMV is the presence of viral inclusions in the mucosal tissue in an area of active inflammation. Endoscopic features alone will not allow one to make the diagnosis of CMV superimposed on IBD.

## 4. Endoscopy for Colorectal Cancer Surveillance

 No review of endoscopy in IBD would be complete without discussing the important role that colonoscopy can play in colorectal cancer surveillance. The American Gastroenterological Association (AGA) has recently published a medical position statement [31] and technical review [32] that comprehensively detail the diagnosis and management of colorectal neoplasia in IBD.

 Although there has been no study to date that has shown a direct mortality benefit from colon cancer screening in IBD with colonoscopy, it is accepted that cancers may be detected earlier and at presumably curable stages. The major gastrointestinal societies (AGA, American College of Gastroenterology, American Society for Gastrointestinal Endoscopy, British Society of Gastroenterology) have put forth guidelines for the recommended starting times and intervals for screening. Because the risk of colonic neoplasia is probably similar in Crohn's colitis as in UC [33], these guidelines should also apply to patients with Crohn's colitis for similar extent, duration, and age of onset of disease. In patients who have had pancolitis for eight years (starting at the time of symptoms), screening should begin at 1-2-year intervals. For left-sided disease, current guidelines from most societies recommend starting screening after 15 years of disease. The exact interval of screening in this latter group of patients is not established and the societies recommend in general every 1-2 years. The British Society of Gastroenterology suggests decreasing the yearly interval of surveillance with each decade of disease (they suggest a screening colonoscopy every 3 years in the second decade of disease, every 2 years in the third decade, and every year in the fourth decade [34]). Societies agree that patients with isolated proctitis probably do not have to be screened for colon cancer.

 There are several factors that need to be taken into consideration when determining the correct screening interval for your patient. Patients who have had treatment refractory or persistent inflammation throughout their IBD history might be at increased risk for progression to neoplasia [35], and one should consider starting surveillance sooner. Whether or not a patient has a personal or family history of adenomatous polyps or colon cancer should also be factored in to the screening commencement time and interval. If a patient has a concurrent diagnosis of primary sclerosing cholangitis (PSC), it is recommended that colorectal cancer screening start at the time of diagnosis of PSC and be continued at yearly intervals. Patients should ideally be in clinical remission at the time of screening colonoscopy so as to minimize confusion between inflammation and dysplasia on histology.

 Dysplasia in the colon of a patient with IBD can be so called “flat dysplasia” that by white light looks indistinguishable from the surrounding tissue. Therefore, the current standard of care for colon cancer screening in IBD includes random 4 quadrant biopsies. These are carried out at regular 10 cm intervals for at least 33 biopsies to achieve optimal sensitivity. All biopsies from each area should be placed in separate, carefully labeled jars. This screening should only be done in areas known to be involved in the past (i.e., the right colon does not need to be surveyed in left-sided UC). A patient that has flat high-grade dysplasia detected in any one area, or at least two areas of flat low-grade dysplasia should be advised colectomy. If a patient has one area of flat low-grade dysplasia detected upon random sampling (and that is verified by a second gastrointestinal pathologist), then a discussion should follow with the patient with regards to colectomy versus further screening colonoscopies at a reduced interval. Several studies have documented an increased risk for development to neoplasia from low-grade dysplasia, and one study found that 4/17 (23.5%) of resected colon specimens for flat low-grade dysplasia harbored advanced neoplasia [36].

**Figure 4:**
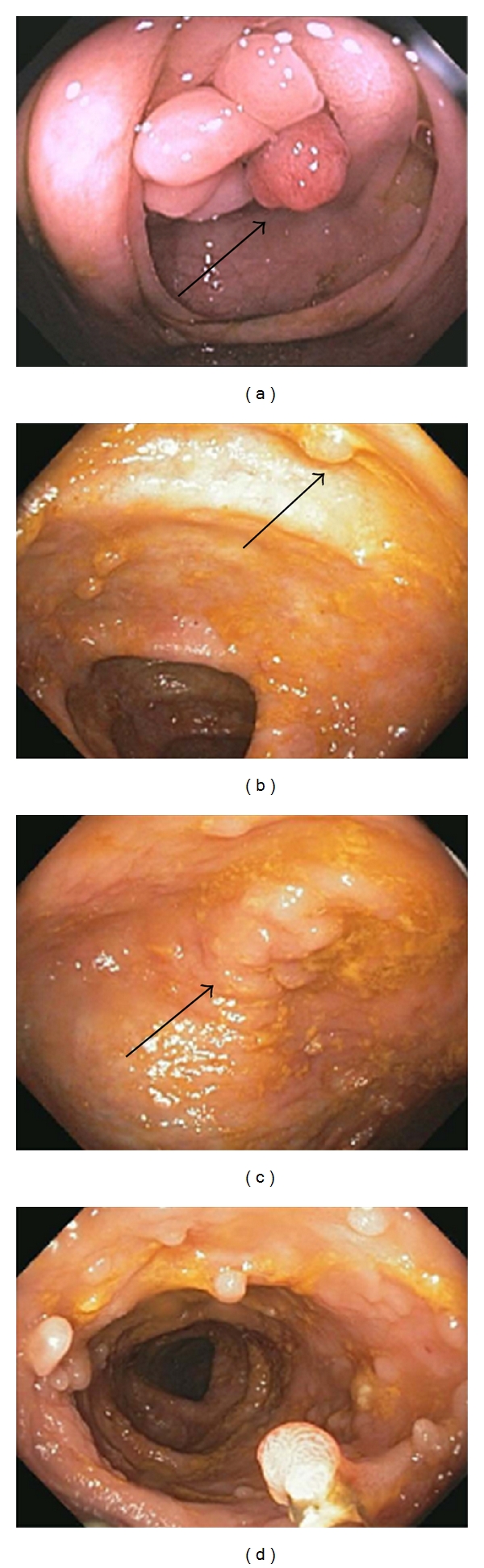
Protruding lesions seen during colon cancer screening in IBD. A patient with left-sided ulcerative colitis was found to have a sporadic adenomatous polyp (black arrow) at the ileocecal valve (a). An adenoma-like DALM (black arrow) in an area of previous inflammation (b) and a non-adenoma-like DALM (black arrow) (c) were identified in 2 patients screened for colon cancer. Pseudopolyps were found in a patient with longstanding ulcerative colitis (d).

 What else should the endoscopist look for during a screening colonoscopy in a patient with IBD? As in a standard screening colonoscopy, careful attention must be placed on all masses, polypoid lesions, and other protruding lesions from the mucosa. As mentioned, strictures need careful attention particularly in UC where a stricture is cancer until proven otherwise. Polyps in noninflamed areas are likely sporadic (Figure 4(a)) and should be treated as in the non-IBD patient. If dysplasia is detected in any protruding lesion in an area of current or previous inflammation, this is referred to as a dysplasia-associated mass or lesion or “DALM.” Presently, this is further classified as an “adenoma-like” DALM or a “non-adenoma-like” DALM. The former lesion is well circumscribed, sessile, or pedunculated, without ulceration or bleeding, and resembles a sporadic adenoma (Figure 4(b)). In contrast, the non-adenoma-like DALM is not well circumscribed, is broad-based and irregular, and is often associated with bleeding or ulceration (Figure 4(c)). On the surveillance colonoscopy, these lesions should be removed in their entirety or biopsied if this is not possible. The area around the base should be biopsied and tattooed. If an adenoma-like DALM is confidently removed and the base is negative for dysplasia, then surveillance may be resumed. It has been shown that adenoma-like lesions arising in the setting of colitis have a benign course [37, 38]. If the area is not completely removed and if there is any evidence of dysplasia at the base or if there is dysplasia detected anywhere else in the colon, then the patient should be advised colectomy. A non-adenoma-like DALM with irregular borders or flat, spreading appearance that is endoscopically unresectable warrants colectomy given the associated high risk of colon cancer [39].

 Inflammatory polyps can be seen at endoscopy in patients with IBD (Figure 4(d)). These are likely benign lesions caused by the healing of erosive inflammation. The histology of pseudopolyps is characteristic, and they should therefore be biopsied or removed if encountered. They appear as polypoid lesions that can be longer in dimension than they are wide. Their presence may interfere with the safe detection of dysplasia and DALMs.

 There have been recent advances in optical methods and dye systems in order to more accurately detect dysplasia. Although these techniques have not been incorporated into recent guidelines, they may allow for targeted biopsies and eliminate the need for random biopsies. The most promising technique to date and one that has been endorsed recently by the Crohn's and Colitis Foundation of America Colon Cancer in IBD Study Group [40] is chromoendoscopy. In this technique, a dye such as methylene blue or indigo carmine is applied to the colon either randomly or selectively to highlight areas of dysplasia. Methylene blue is an absorptive dye that is taken up by normal tissue but not dysplastic tissue, and indigo carmine is a contrast dye that pools in areas of abnormal dysplastic tissue. Several recent studies have shown that chromoendoscopy increases the sensitivity for the detection of dysplasia. A recently published meta-analysis calculated a pooled incremental yield of chromoendoscopy over white light for dysplasia detection. They were able to find that in the 6 studies of 1277 patients, chromoendoscopy was significantly better than white light for the detection of dysplasia in colonic IBD [41]. In the technical review and position statement published by the AGA, the authors concluded that targeted biopsies using chromoendoscopy performed by endoscopists experienced in this technique is a reasonable screening alternative to the random sampling of colon using standard white light [31, 32]. In the AGA Institute technology assessment on image-enhanced endoscopy (IEE), the authors conclude that IEE may increase the yield of detection of dysplasia and as such is recommended for patients with long-standing UC [42]. Other techniques that are currently under study but are not yet proven for widespread clinical use include confocal microendoscopy and narrow-band imaging.

## 5. Endoscopic Interventions in IBD

 A clinical consequence of many phenotypes of Crohn's disease is stricturing of the intestine. This most commonly occurs at a surgical anastomosis, in the terminal ileum, at the ileocecal valve, and in the colon. Because it is essential to conserve bowel in order to prevent problems such as short gut syndrome, the idea of dilation of strictures using endoscopy is attractive. Studies have shown that endoscopic balloon dilation can be performed safely with minimal complications and can provide years of symptom and surgery-free life [43]. One prospective study showed that the length and location of a stricture may predict the long-term success of the dilation [44]. Of the 55 patients that underwent dilation of 74 symptomatic strictures, only 24% of these patients ultimately required surgery after a median 44 month followup. These strictures were on average 7.5 cm compared with 2.5 cm for the patients that did not require surgery and were all located in the terminal ileum. In the largest series published to date [45], 237 dilations were performed on 138 patients and followed for a median 5.8 years (5% complication rate, 97% immediate success rate, 84% were anastomotic strictures, all strictures were <5 cm in length). Because of recurrent symptoms, 46% of these patients required subsequent dilation, and 24% required surgery. Interestingly, neither the degree of clinical activity of inflammation at the time of dilation as assessed by CRP and endoscopic disease activity, nor the concomitant medical therapy following dilation influenced future need for dilation or surgery. There are reports as well of endoscopic dilation using double-balloon enteroscopy for symptomatic small intestinal strictures that are out of reach of the standard upper endoscope [46].

## 6. Conclusions

 Presented in this review are the many ways in which endoscopy is used in the diagnosis and management of IBD patients. Current research is focusing on the role of mucosal healing as assessed by endoscopy as an endpoint for medical therapy and in postoperative surveillance. The future is promising for cancer and dysplasia surveillance with newer optical equipment and dyes to allow for targeted biopsies. Overall, the diagnosis and management of IBD still starts with a careful history and physical exam. In the appropriately selected patient, endoscopy can provide valuable clinical information to supplement or solidify the clinical impression. The role of endoscopy in IBD is ever evolving and is an important target of clinical education and research.

## References

[1] Leighton JA, Shen B, Baron TH (2006). ASGE guideline: endoscopy in the diagnosis and treatment of inflammatory bowel disease. *Gastrointestinal Endoscopy*.

[2] Waye JD (1990). Endoscopy in inflammatory bowel disease: indications and differential diagnosis. *Medical Clinics of North America*.

[3] Pera A, Bellando P, Caldera D (1987). Colonoscopy in inflammatory bowel disease. Diagnostic accuracy and proposal of an endoscopic score. *Gastroenterology*.

[4] Joo M, Odze RD (2010). Rectal sparing and skip lesions in ulcerative colitis: a comparative study of endoscopic and histologic findings in patients who underwent proctocolectomy. *American Journal of Surgical Pathology*.

[5] Schroeder KW, Tremaine WJ, Ilstrup DM (1987). Coated oral 5-aminosalicylic acid therapy for mildly to moderately active ulcerative colitis: a randomized study. *The New England Journal of Medicine*.

[6] Zwas FR, Bonheim NA, Berken CA, Gray S (1994). Ileoscopy as an important tool for the diagnosis of Crohn’s disease: a report of seven cases. *Gastrointestinal Endoscopy*.

[7] Haskell H, Andrews CW, Ready SI (2005). Pathologic features and clinical significance of "backwash" ileitis in ulcerative colitis. *American Journal of Surgical Pathology*.

[8] Coremans G, Rutgeerts P, Geboes K, Van den Oord J, Ponette E, Vantrappen G (1984). The value of ileoscopy with biopsy in the diagnosis of intestinal Crohn's disease. *Gastrointestinal Endoscopy*.

[9] Bernstein CN, Shanahan F, Anton PA, Weinstein WM (1995). Patchiness of mucosal inflammation in treated ulcerative colitis: a prospective study. *Gastrointestinal Endoscopy*.

[10] Glickman JN, Bousvaros A, Farraye FA (2004). Pediatric patients with untreated ulcerative colitis may present initially with unusual morphologic findings. *American Journal of Surgical Pathology*.

[11] Nasseri Y, Melmed G, Wang HL, Targan S, Fleshner P (2010). Rigorous histopathological assessment of the colectomy specimen in patients with inflammatory bowel disease unclassified does not predict outcome after ileal pouch-anal anastomosis. *American Journal of Gastroenterology*.

[12] D'Haens G, Geboes K, Peeters M, Baert F, Ectors N, Rutgeerts P (1997). Patchy cecal inflammation associated with distal ulcerative colitis: a prospective endoscopic study. *American Journal of Gastroenterology*.

[13] Mantzaris GJ, Hatzis A, Archavlis E (1995). The role of colonoscopy in the differential diagnosis of acute, severe hemorrhagic colitis. *Endoscopy*.

[14] Tedesco FJ, Hardin RD, Harper RN, Edwards BH (1983). Infectious colitis endoscopically simulating inflammatory bowel disease: a prospective evaluation. *Gastrointestinal Endoscopy*.

[15] Bhargava DK, Tandon HD, Chawla TC (1985). Diagnosis of ileocecal and colonic tuberculosis by colonoscopy. *Gastrointestinal Endoscopy*.

[16] Surawicz CM, Haggitt RC, Husseman M, McFarland LV (1994). Mucosal biopsy diagnosis of colitis: acute self-limited colitis and idiopathic inflammatory bowel disease. *Gastroenterology*.

[17] Stolte M, Hartmann FO (2010). Misinterpretation of NSAID-induced Colopathy as Crohn’s disease. *Zeitschrift fur Gastroenterologie*.

[18] Wagtmans MJ, van Hogezand RA, Griffioen G, Verspaget HW, Lamers CB (1997). Crohn’s disease of the upper gastrointestinal tract. *Netherlands Journal of Medicine*.

[19] Tobin JM, Sinha B, Ramani P, Saleh ARH, Murphy MS (2001). Upper gastrointestinal mucosal disease in pediatric Crohn disease and ulcerative colitis: a blinded, controlled study. *Journal of Pediatric Gastroenterology and Nutrition*.

[20] Valdez R, Appelman HD, Bronner MP, Greenson JK (2000). Diffuse duodenitis associated with ulcerative colitis. *American Journal of Surgical Pathology*.

[21] Leighton JA (2010). The Role of Endoscopic Imaging of the Small Bowel in Clinical Practice. *American Journal of Gastroenterology*.

[22] Pineton De Chambrun G, Peyrin-Biroulet L, Lémann M, Colombel J (2010). Clinical implications of mucosal healing for the management of IBD. *Nature Reviews Gastroenterology and Hepatology*.

[23] Lichtenstein GR, Rutgeerts P (2010). Importance of mucosal healing in ulcerative colitis. *Inflammatory Bowel Diseases*.

[24] Rutgeerts P, Feagan BG, Lichtenstein GR (2004). Comparison of scheduled and episodic treatment strategies of infliximab in Crohn’s disease. *Gastroenterology*.

[25] Regueiro M, Rodemann J, Kip KE (2011). Physician assessment of ulcerative colitis activity correlates poorly with endoscopic disease activity. *Inflammatory Bowel Diseases*.

[26] Rutgeerts P, Geboes K, Vantrappen G, Beyls J, Kerremans R, Hiele M (1990). Predictability of the postoperative course of Crohn’s disease. *Gastroenterology*.

[27] Rutgeerts P, Van Assche G (2008). What is the role of endoscopy in the postoperative management of Crohn's disease?. *Inflammatory Bowel Diseases*.

[28] Shen B, Achkar JP, Lashner BA (2001). Endoscopic and histologic evaluation together with symptom assessment are required to diagnose pouchitis. *Gastroenterology*.

[29] Shen B, Fazio VW, Remzi FH (2005). Comprehensive evaluation of inflammatory and noninflammatory sequelae of ileal pouch-anal anastomoses. *American Journal of Gastroenterology*.

[30] Ben-Horin S, Margalit M, Bossuyt P (2010). Prevalence and clinical impact of endoscopic pseudomembranes in patients with inflammatory bowel disease and Clostridium difficile infection. *Journal of Crohn’s and Colitis*.

[31] Farraye FA, Odze RD, Eaden J, Itzkowitz SH (2010). AGA medical position statement on the diagnosis and management of colorectal neoplasia in inflammatory bowel disease. *Gastroenterology*.

[32] Farraye FA, Odze RD, Eaden J, Itzkowitz SH (2010). AGA technical review on the diagnosis and management of colorectal neoplasia in inflammatory bowel disease. *Gastroenterology*.

[33] Friedman S, Rubin PH, Bodian C, Harpaz N, Present DH (2008). Screening and surveillance colonoscopy in chronic Crohn’s colitis: results of a surveillance program spanning 25 years. *Clinical Gastroenterology and Hepatology*.

[34] Eaden JA, Mayberry JF (2002). Guidelines for screening and surveillance of asymptomatic colorectal cancer in patients with inflammatory bowel disease. *Gut*.

[35] Rutter M, Saunders B, Wilkinson K (2004). Severity of inflammation is a risk factor for colorectal neoplasia in ulcerative colitis. *Gastroenterology*.

[36] Ullman T, Croog V, Harpaz N, Sachar D, Itzkowitz S (2003). Progression of Flat Low-Grade Dysplasia to Advanced Neoplasia in Patients with Ulcerative Colitis. *Gastroenterology*.

[37] Engelsgjerd M, Farraye FA, Odze RD (1999). Polypectomy may be adequate treatment for adenoma-like dysplastic lesions in chronic ulcerative colitis. *Gastroenterology*.

[38] Odze RD, Farraye FA, Hecht JL, Hornick JL (2004). Long-term follow-up after polypectomy treatment for adenoma-like dysplastic lesions in ulcerative colitis. *Clinical Gastroenterology and Hepatology*.

[39] Blackstone MO, Riddell RH, Rodgers BH, Levin B (1981). Dysplasia-associated lesion or mass (DALM) detected by colonoscopy in long-standing ulcerative colitis: an indication for colectomy. *Gastroenterology*.

[40] Itzkowitz SH, Present DH, Binder V (2005). Consensus conference: colorectal cancer screening and surveillance in inflammatory bowel disease. *Inflammatory Bowel Diseases*.

[41] Subramanian V, Mannath J, Ragunath K, Hawkey CJ (2011). Meta-analysis: the diagnostic yield of chromoendoscopy for detecting dysplasia in patients with colonic inflammatory bowel disease. *Alimentary Pharmacology and Therapeutics*.

[42] Kaltenbach T, Sano Y, Friedland S, Soetikno R (2008). American gastroenterological association (AGA) institute technology assessment on image-enhanced endoscopy. *Gastroenterology*.

[43] Scimeca D, Mocciaro F, Cottone M (2011). Efficacy and safety of endoscopic balloon dilation of symptomatic intestinal Crohn’s disease strictures. *Digestive and Liver Disease*.

[44] Mueller T, Rieder B, Bechtner G, Pfeiffer A (2010). The response of Crohn’s strictures to endoscopic balloon dilation. *Alimentary Pharmacology and Therapeutics*.

[45] Thienpont C, D’Hoore A, Vermeire S (2010). Long-term outcome of endoscopic dilatation in patients with Crohn’s disease is not affected by disease activity or medical therapy. *Gut*.

[46] Hirai F, Beppu T, Sou S, Seki T, Yao K, Matsui T (2010). Endoscopic balloon dilatation using double-balloon endoscopy is a useful and safe treatment for small intestinal strictures in crohn’s disease. *Digestive Endoscopy*.

